# Bipedal locomotion in *Octopus vulgaris*: A complementary observation and some preliminary considerations

**DOI:** 10.1002/ece3.7328

**Published:** 2021-03-05

**Authors:** Piero Amodio, Noam Josef, Nadav Shashar, Graziano Fiorito

**Affiliations:** ^1^ Department of Biology and Evolution of Marine Organisms Stazione Zoologica Anton Dohrn Napoli Italy; ^2^ Department of Life Sciences Ben‐Gurion University Eilat Israel

**Keywords:** behavioral flexibility, bipedal, cephalopods, locomotion, Octopus, walking

## Abstract

Lacking an external shell and a rigid endoskeleton, octopuses exhibit a remarkable flexibility in their movements. Bipedal locomotion is perhaps the most iconic example in this regard. Until recently, this peculiar mode of locomotion had been observed only in two species of tropical octopuses: *Amphioctopus marginatus* and *Abdopus aculeatus*. Yet, recent evidence indicates that bipedal walking is also part of the behavioral repertoire of the common octopus, *Octopus vulgaris*. Here we report a further observation of a defense behavior that encompasses both postural and locomotory elements of bipedal locomotion in this cephalopod. By highlighting differences and similarities with the other recently published report, we provide preliminary considerations with regard to bipedal locomotion in the common octopus.

## Graphical abstract

Here we report a further observation of a defense behavior that encompasses both postural and locomotory elements of bipedal locomotion in *Octopus vulgaris*. Our observation complements previous reports by showing that *O. vulgaris* can flexibly incorporate postural and locomotory components of bipedal walking amid heterogeneous displacement sequences.

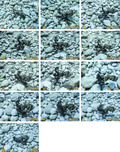

## 1 INTRODUCTION

Invertebrate molluscs typically show a very limited flexibility in their movements. A thick external shell dramatically constraints locomotory patterns in most members of this group. In octopuses, however, the molluscan shell is absent. The bodies of these animals lack of any rigid structure, with the exception of a cartilaginous “skull” and a chitinous beak that are located in the head (Wells, 1978). Furthermore, in octopuses the molluscan foot was transformed partly into a set of eight suckered appendages and partly into a mobile funnel that allows the fast ejection of water from the mantle (Shigeno et al., 2008). As result of these adaptations, octopuses exhibit an extraordinary versatility of movements and postures. For instance, each arm can be elongated, shortened, bent, or twisted independently from the others and with virtually infinite degrees of freedom (Sumbre et al., 2001), adopting different postures, maneuvres, and locomotory patterns (review in Borrelli et al., 2006; Mather, 1998). At the same time, the mantle can assume shapes as different as a swelled and vertically oriented sack (i.e., mantle ballooning, review in Borrelli et al., 2006) or a flattened sack oriented parallel to the substrate (i.e., mantle rounded, Packard & Sanders, 1971), to mention some.

Locomotion is equally diverse in octopuses. These molluscs can crawl across the substrate via coordinated pushing and pulling actions performed by arms and suckers, swim forward or backward by expelling expel water jets from the siphon, and even walk bipedally on two arms (Huffard, 2006; for a review, see Hanlon & Messenger, 2018; Levy & Hochner, 2017). The latter is an extremely sophisticated mode of locomotion, from a biomechanical perspective.

Bipedal locomotion in the octopus is not produced by the action of antagonistic muscles against a rigid skeleton as in vertebrates; rather, it is achieved through the concerted action of differently oriented components (Huffard et al., 2005) within a muscular hydrostatic system (Kier & Smith, 1985). The differential contraction of transverse, longitudinal, and oblique bundle of muscles allows the octopus to stiffen and relax different segments of the same arm, thereby supporting bipedal walking (Huffard et al., 2005).

While engaging into bipedal gates (also termed rolling gates), typically through the action of arms IV (Huffard et al., 2005), octopuses may assume deceptive appearances. *Abdopus aculeatus* often exhibits a highly disruptive Flamboyant‐like body pattern (sensu Packard & Sanders, 1969, 1971) with spread and helically coiled arms tips (arms I‐III), mottled coloration and raised papillae (Huffard, 2006; Huffard et al., 2005). Note, however, that body patterns expressed by this species during bipedal locomotion are variable and can encompass also arms I‐III held close to the body and a striped pattern (dark coloration with pale medial stripes, Huffard, 2006). On the other hand, *Amphioctopus marginatus* displays a more rounded and homogenous appearance, with arms I‐III tucked on the side or below the body, usually exhibiting smooth skin and brownish coloration with dark stripes on the arms (Huffard et al., 2005). The postural, chromatic, and textural features expressed by these octopuses might resemble the appearance of distinctive elements of their environment (e.g., detached algae in *A. aculeatus*, coconut shell in *A. marginatus*), such that when the locomotory component of the bipedal walking is also taken into account, octopuses seem to impersonate a loose environmental element dragged around by the current. As a result, predators’ ability to form a specific search image for these species may be hindered (Huffard, 2006). Thus, bipedal locomotion in octopus may constitute an anti‐predatory strategy laying in between crypis and flight response, respectively, representing, the primary and (one possible) secondary defense tactics (Huffard, 2006).

In addition, the fact that arms I‐III are typically not involved in the locomotion, and thus free for other purposes, may provide an added value in terms of defense for the octopus (e.g., “arm‐slap,” Woods, 1965; “punching,” Sampaio et al., 2020).

Until recently, bipedal locomotion in octopus had been observed only in the two aforementioned species. Yet, new evidence indicates that this peculiar form of locomotion may also be part of the behavioral repertoire of one of the most iconic cephalopod, the common octopus (*Octopus vulgaris*).

Formerly described as a single taxonomic unit with a cosmopolitan distribution (e.g., Norman, 2000), *O. vulgaris* is now considered a group encompassing multiple cryptic species (Amor et al., 2019; De Luca et al., 2014, 2016), including *O. vulgaris* sensu *stricto* (Mediterranean and eastern North Atlantic) and *O. vulgaris* Type III (South Africa). In addition, some populations that were initially considered part of *O. vulgaris* species complex are currently treated as distinct species (e.g., *Octopus sinensis,* Gleadall, 2016).

In the Atlantic waters of Spain, Hernández‐Urcera et al. (2020) observed a small‐sized *O. vulgaris* (sensu *stricto*) performing a defense behavior that has been classified as bipedal locomotion. While keeping contact with the bottom, the octopus engaged in a backward rolling gate mainly through the action of arms IV. However, arms III and even II also appear to be involved in the locomotion (see Video S1 in Hernández‐Urcera et al., 2020), such that it is possible that the observation might represent a mixture of bipedal and multi‐arm‐walking (sensu Huffard, 2006). In the first part of the displacement, arms I were raised with coiled tips. Yet subsequently, these arms were lowered and held close to arms II and III in a curled posture with partially spread interbrachial web. Throughout the displacement, the octopus expressed a disruptive appearance with chromatic and textural components of the Flamboyant body pattern.

**FIGURE 1:**
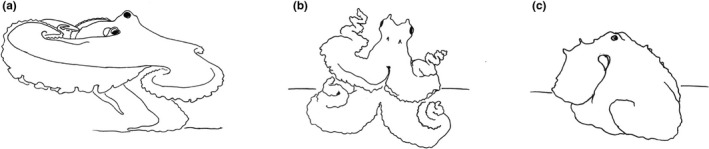
Visual description of the patterns exhibited by *Octopus vulgaris* (Type III) during bipedal and/or multi‐arm walking. The sketches were drawn from still images extracted from the documentary “My octopus teacher” (Ehrlich & Reed, 2020)

Additional evidence was also collected by observing *O. vulgaris* type III in the wild. The recent documentary “My octopus teacher” (Ehrlich & Reed, 2020) captured multiple instances of bipedal walking in South African waters. Interestingly, different body patterns were expressed during locomotion (Table 1; Figure 1). For instance, in one case an octopus directed water jets toward the camera while exhibiting a dark and smooth skin, with arms I‐III curved and interbrachial web maximally spread (Figure 1a).

**TABLE 1:** Patterns exhibited by *Octopus vulgaris* species complex during bipedal and/or multi‐arm walking

*O. vulgaris* species	Pattern	Main chromatic (c), textural (t) and postural (p) components			References	Visual description (see Figures 1, 2)
		(c)	(t)	(p)		
Sensu *stricto*	Flamboyant	Frontal white spots, mantle white spots, white papillae, arm white spots, arm bars	Rough skin, long mantle papillae, long head papillae, papillae on side, back fin	Arms I twisted, arms II tucked in and curled in the distal part, mantle ogive	Hernández‐Urcera et al. (2020) This study	Figure 2
Type III	Ground Dark Brown (?)	Dark mottle	Smooth skin, long mantle papillae	Arms I, II, III spread, interbrachial web spread, funnel directed toward stimulus	Ehrlich and Reed (2020)	Figure 1a
Type III	Undescribed^a^	Uniform reddish brown	Smooth skin, long mantle papillae, long head papillae	Arms I, II, III coiled	Ehrlich and Reed (2020)	Figure 1b
Type III	Moving Rock^b^	“Cryptic brownish with dark arms”	Rough skin, long mantle papillae	Arms I, II, III held close or below the body, head flattened, crouched body	Ehrlich and Reed (2020)	Figure 1c

Note

For definitions see (Borrelli et al., 2006)

^a^ Note that the postural components of this pattern are remarkably similar to a posture that has been described in *Abdopus aculeatus* (cfr. fig. 4a, Huffard, 2006).

^b^ The textural and postural components of this appearance closely resemble those expressed by *Octopus cyanea* in the “Moving Rock,” a cryptic body pattern through which an octopus assumes the appearance of a rounded coral/rock and slowly moves on the substrates by using the tips of its arms (Hanlon et al., 1999).

Here, we report a further observation of a defense response in *O. vulgaris* sensu *stricto* from Mediterranean Sea that encompasses postural and locomotory elements of bipedal walking (Video S1).

## 2 MATERIAL AND METHODS

### 2.1 Study animal and site

On 10 May 2010, in the morning, we were conducting a SCUBA diving survey at Capri (Italy), to collect data for a study on octopus camouflage abilities (Josef et al., 2012). The site is characterized by a white pebble substrate with intersperse patches of seagrass (*Posidonia oceanica*). We spotted an *O. vulgaris* in the open at a depth of approximately 5 m. The exact size of the individual is not known, but we estimated that it has weighted 100–150 g. The animal had a missing part of a posterior arm (arm III Left). We started to observe the octopus and video‐recorded its behavioral response (Video S1).

### 2.2 Analysis

To describe the observed behavioral response, we conducted a frame‐by‐frame analysis of the video on Avidemux (ver. 2.7.6; http://fixounet.free.fr/avidemux/). The chromatic, textural, postural, and locomotory elements exhibited by the animal were categorized based on definitions by Packard and Sanders (1971) and those reviewed in Borrelli et al. (2006).

## 3 RESULTS AND DISCUSSION

**FIGURE 2:**
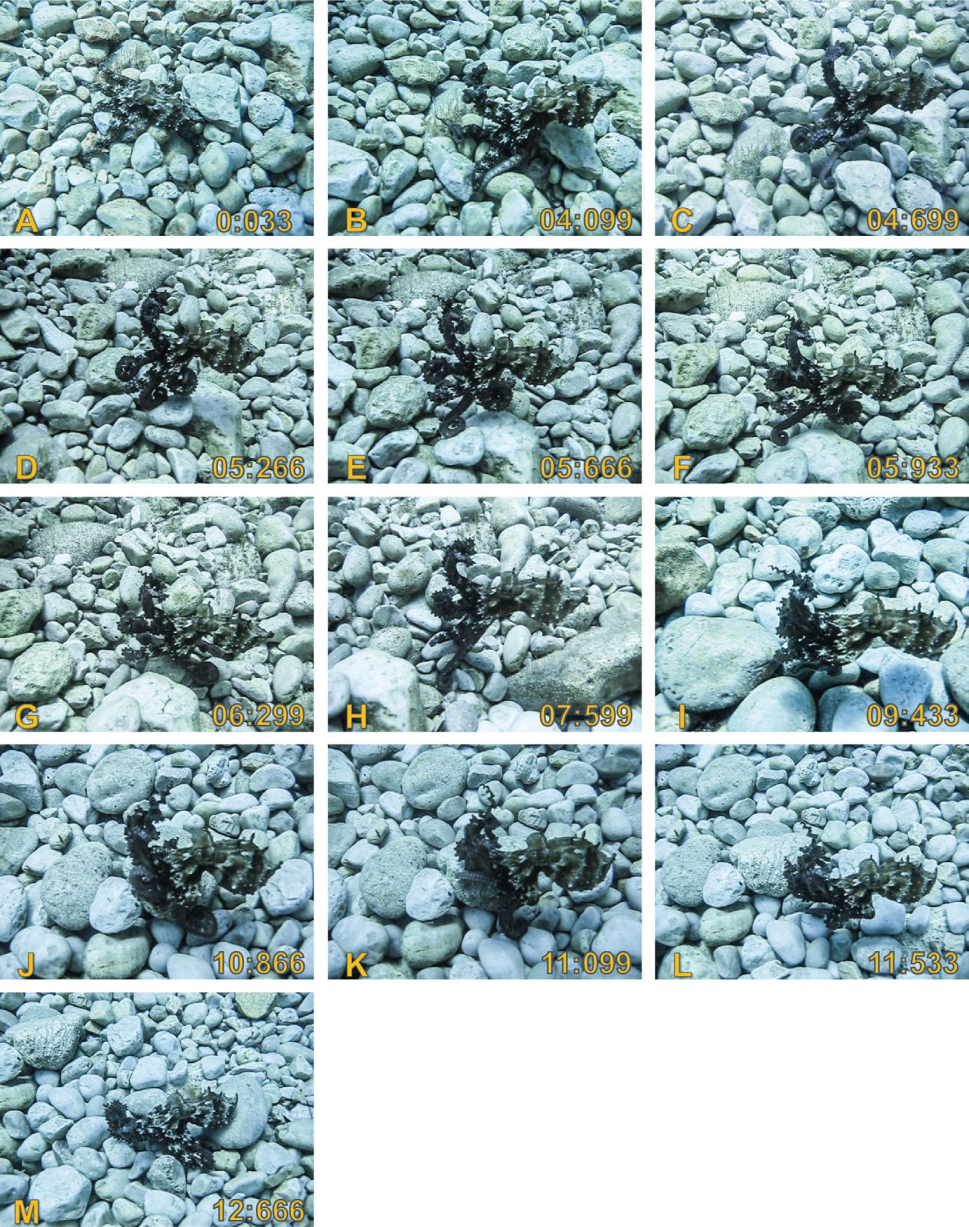
Still images describing the behavioral sequence. See main text and Video S1 for details

The animal reacted to our presence by remaining still on the bottom and exhibiting a disruptive body pattern (Figure 2a). The following chromatic (c), textural (t), and postural (p) elements were expressed, c: frontal white spots, mantle white spots, white papillae, arm white spots, arm bars; t: long mantle papillae, long head papillae, papillae on side, back fin; p: arms loose, mantle ogive (Borrelli et al., 2006; Packard & Sanders, 1971). At this stage, water flushes from the siphon were also directed toward us (i.e., funnel directed toward external stimulus, Packard & Sanders, 1971).

Next, the animal gradually raised its arms from the substrate and assumed a Flamboyant body pattern with arms I twisted (Figure 2b) and arms II tucked in and curled in the distal part (Figure 2c; Borrelli et al., 2006). These postural changes co‐occurred with the start of a backward displacement. While lifting up the last arm from the substrate (arm IV Right), the octopus pointed the siphon downward and flushed a water jet (Figure 2c), thus “hopping” backward and then landing on one arm (arm III R; Figure 2d).

From a biomechanical perspective, this is an interesting sequence of movements because it involved two posterior arms of the same side—arm IV R to lift‐off (Figure 2c) and arm III R to land (Figure 2d)—rather than one right arm and one left arm as typically observed in bipedal locomotion (Huffard et al., 2005). Next, the distal part of arm III R—which was tucked in and curled—was progressively “unrolled,” thus pushing the whole animal further back (Figure 2d–f). Notably, the postural (e.g., coiled tips in arms I; arms II and IV tucked in and curled) and locomotory components exhibited in this sequence are remarkably similar to those described for bipedal walking in *A. aculeatus* (see: figure 1 in Huffard et al., 2005; figure 2d in Huffard, 2006). Nevertheless, this displacement does not qualify as a bipedal walk because the octopus briefly lost contact with the substrate (Figure 2f) and then landed with multiple arms (arms III R, arms IV; Figure 2g).

Next, the animal stood on arms IV (Figure 2h)—in a posture that resemble the Flamboyant body pattern in *Octopus bimaculoides* (see figure 10 in Forsythe & Hanlon, 1988)—before to initiate a jet‐propelled backward swimming (Figure 2i). This displacement was followed by a bipedal walk: the octopus (a) landed on arm IV R (Figure 2j); (b) gradually “unrolled” arm IV L to contact the bottom (Figure 2j,k); (c) used arm IV R to push the body obliquely; and (d) lifted up arm IV R (Figure 2l). Finally, the animal lifted up arm IV L as well and performed a jet‐propelled “hop,” before to land on the bottom with multiple arms (Figure 2m).

Our observation complements the report by Hernández‐Urcera et al. (2020) in two respects. First, it shows that *O. vulgaris*—as *A. aculeatus* (Huffard, 2006)—can be employ bipedal walks to perform oblique displacements (Figure 2j–l), not only backward displacements as previously reported. Second, our observation indicates that *O. vulgaris* can flexibly incorporate postural and locomotory components of bipedal walking amid heterogeneous displacement sequences.

Whereas Hernández‐Urcera et al. (2020) recorded a continuous rolling gate encompassing a number of consecutive bipedal (and/or multi‐arm) walks, we observed a rather diverse displacement sequence that involved smooth transitions among distinct locomotory patterns, namely jet‐propelled hopping (04:566–04:999, 11:633–11:999), nonjet‐propelled hopping (04:999–06:166), and backward swimming (07:733–10:033), in addition to bipedal walking (10:066–11:599). Interestingly, the postural and locomotory components of bipedal walking were not exhibited only during the bipedal walk (Figure 2j–l) but also during hopping (Figure 2d–g), similarly to what has been observed in *A. aculeatus* by Huffard (2006).

It should be noted that nonjet‐propelled hopping in the octopus might bear some similarities with *underwater punting* (Chellapurath et al., 2020; Martinez et al., 1998), a type of locomotion described in crabs. In both cases, a thrust—generated by the limb(s) acting against the substrate—allows the body to displace by gliding away in the water. Considering that octopuses can generate the thrust force not only through the muscular action of the arms but also through jet‐propulsion, it would be intriguing to characterize the kinematics of hopping in these animals, perhaps in comparison with *underwater punting* by crabs (e.g., Chellapurath et al., 2020) and/or bipedal locomotion in octopus (Huffard et al., 2005). This may be a particularly interesting comparison given that octopuses are only slightly negatively buoyant.

The differences in locomotory patterns between the observation reported here and the one made by Hernández‐Urcera et al. (2020) are intriguing given that the two behaviors were defensive responses triggered by the same stimuli (i.e., SCUBA divers). It is possible that the lack of a part of arm III L in our octopus might have to some extent limited the locomotory ability of the animal, thereby favoring jet‐propelled hopping and swimming over continuous rolling gates.

Alternatively, it is also possible that specific features of the substrates might have played a role. The observation by Hernández‐Urcera et al. (2020), as well as the reports of bipedal locomotion in other species (Huffard, 2006; Huffard et al., 2005), took place in a sandy bottom environment. In contrast, the behavior we have observed was performed on a pebble substrate that, being more uneven, might have impaired octopus’ ability to perform quick displacements through continuous rolling gaits. Future research is needed in order to test these hypotheses.

In parallel, the observation reported here and the one made by Hernández‐Urcera et al. (2020) also share some important features. In particular, both reports involved a small‐sized *O. vulgaris*. Note that this is a fair, although crude, categorization given that this cephalopod can exceed more than five kilograms of body weight (Jereb et al., 2014). Further, the appearances assumed by the animals during locomotion encompassed chromatic, textural, and postural components of the Flamboyant body pattern (e.g., frontal white spots, rough skin, arms I twisted; Table 1).

According to Packard and Sanders (1971), the Flamboyant body pattern in *O. vulgaris* is a response to disturbance that is specific to small‐sized animals; this “immature” response is gradually replaced by the Dymantic body pattern in larger size individuals. Building on this, it may be reasonable to hypothesize that if bipedal walking in *O. vulgaris* is predominantly expressed together with the Flamboyant, then this locomotory pattern should be restricted to small‐sized individuals. Alternatively, it has been proposed that bipedal locomotion might be restricted to small octopuses due to the physical constraints imposed by larger body mass (Hernández‐Urcera et al., 2020). However, given the variability observed in *O. vulgaris* type III with regard to the body size of the “walker” and body pattern expressed during bipedal locomotion (Ehrlich & Reed, 2020), these considerations should be taken with caution.

In summary, our observation provides further evidence that *O. vulgaris* is capable of bipedal walking, thereby enriching the recent report by Hernández‐Urcera et al. (2020). Yet, future research will be essential to gain further insight into this issue.

The approach used by Huffard (2006) could be replicated in *O. vulgaris* in order to characterize the variability expressed by this cephalopod in terms of body patterns exhibited during locomotion and body size of the “walker.” Systematic observations would also allow to clarify to what extent the features of the substrate (e.g., sandy vs. pebble bottom) and/or morphological factors (i.e., missing arm) might influence octopus’ ability to walk bipedally. Finally, considering that cephalopods are known for adjusting their anti‐predatory according to the hunting strategies of predators (Langridge et al., 2007; Staudinger et al., 2011; for a review, see Amodio et al., 2020), it would be particularly interesting to investigate whether bipedal locomotion is flexibly exhibited depending on the kind of threat, or ecological context, and whether octopuses are more likely to rely on this locomotory strategy to achieve crypsis while moving (Borrelli et al., 2006; Hanlon et al., 1999; Van Heukelem, 1983), in response to a visual predator relatively to a chemosensory predator.

## CONFLICT OF INTEREST

Authors declare that they have no conflict of interest.

## AUTHOR CONTRIBUTIONS

**Piero Amodio:** Conceptualization (lead); formal analysis (lead); funding acquisition (equal); investigation (equal); visualization (lead); writing – original draft (lead); writing – review and editing (equal). **Noam Josef:** Funding acquisition (equal); investigation (equal). **Nadav Shashar:** Funding acquisition (equal); investigation (equal); writing – review and editing (equal). **Graziano Fiorito:** Conceptualization (supporting); funding acquisition (equal); writing – review and editing (equal).

## Supporting information

Video S1

## Data Availability

Video of the defense response by *Octopus vulgaris* in Capri, Italy: Supporting Information (Video S1).
